# Embolization by Gelatin Sponge for Tract Closure After Inappropriate Procedure of Percutaneous Endoscopic Gastrostomy: Safe Management of Hepatic Injury After Transhepatic Placement

**DOI:** 10.1002/deo2.70255

**Published:** 2025-11-26

**Authors:** Hiroshi Yukimoto, Kohsaku Ohnishi, Koki Nagano, Naoko Hayata, Rie Ito, Motohiro Hirao, Yasuhiro Nakaya, Masanori Tsujie, Atsushi Hosui, Naoki Hiramatsu

**Affiliations:** ^1^ Department of Diagnostic Radiology Osaka Rosai Hospital Sakai Osaka Japan; ^2^ Department of Gastroenterology and Hepatology Osaka Rosai Hospital Sakai Osaka Japan; ^3^ Department of Otolaryngology and Head and Neck Surgery Osaka Rosai Hospital Sakai Osaka Japan; ^4^ Department of Gastroenterological Surgery Osaka Rosai Hospital Sakai Osaka Japan

**Keywords:** complications, embolization, gelatin sponge, hepatic injury, percutaneous endoscopic gastrostomy

## Abstract

Placement of percutaneous endoscopic gastrostomy (PEG) is generally safe and well‐tolerated. Transhepatic insertion of PEG tubes is an extremely rare but serious complication. Optimal management strategies for safe removal of PEG tubes remain unclear. A 70‐year‐old male with hypopharyngeal cancer underwent PEG placement prior to chemoradiotherapy. The patient experienced mild puncture site pain. Progressive elevation of inflammatory markers prompted computed tomography (CT) imaging on postoperative day 2, which revealed inadvertent transhepatic catheter placement through the lateral segment. Minimally invasive removal was prioritized over surgical management to enable continuation of chemoradiotherapy. To prevent bleeding and biliary leakage, gelatin sponge (GS) was embolized for tract closure. A pull‐through technique was established for emergency tract access if massive bleeding occurred. After removal of the tube, embolic material was injected under fluoroscopic and endoscopic guidance. The gastric entry site was closed with endoscopic clips. Serial CT showed no bleeding or biliary leakage, and the patient recovered uneventfully and completed chemoradiotherapy without treatment delays. Embolization of GS represents a safe and effective technique for the removal of a transhepatic PEG tube. This minimally invasive approach successfully prevented serious complications in this rare case of PEG‐related hepatic injury.

## 1 Introduction

Percutaneous endoscopic gastrostomy (PEG) is a widely accepted procedure for long‐term enteral nutrition [1]. While generally safe, it might have serious complications without proper technique or adherence to anatomical landmarks. Inadvertent transhepatic insertion is one of the most concerning complications, which is extremely rare with limited reported cases [2, 3].

Transhepatic PEG placement typically results when the lateral segment is interposed between the gastric and abdominal wall [2]. Adequate transillumination is essential during PEG procedures, particularly in cases of hepatomegaly or previous abdominal surgery, to minimize the risk of organ injury [1, 4, 5]. The clinical symptoms vary significantly, with approximately 31% of cases remaining asymptomatic and discovered incidentally, while others may present with life‐threatening complications, including hemorrhage, biliary leakage, or hepatic abscess formation [2].

Strategies to manage transhepatic PEG tubes remain controversial due to limited case reports. Conservative management has been successful in approximately 69% of cases, while surgical intervention is required in 31% of patients, particularly those with hemodynamic instability or active bleeding [2]. However, optimal approaches are not well established to remove the PEG tube safely without complications. We present a novel case utilizing gelatin sponge (GS) to embolize for tract closure, demonstrating a minimally invasive alternative to surgical management.

## 2 Case Presentation

A 70‐year‐old male with locally advanced hypopharyngeal cancer (cT3N3b) was scheduled for concurrent chemoradiotherapy. The patient had no history of hepatomegaly, previous abdominal surgery, coagulopathy, or use of anticoagulant and antiplatelet medications. Preoperative laboratory studies, including complete blood count and coagulation parameters, were within normal limits. Preoperative computed tomography (CT) showed no hepatomegaly or anatomical variants contraindicating PEG placement. Anticipating nutritional difficulties during treatment, PEG placement was planned for enteral access. During the procedure using the introducer technique with a 24‐Fr gastrostomy catheter (Ideal PEG Kit; Olympus, Tokyo, Japan), standard safety protocols, including finger sign confirmation and adequate transillumination, were performed. However, the patient became agitated despite sedation, and the procedure was completed with additional sedation and physical restraints.

The initial postoperative course appeared uneventful with routine monitoring, although the patient experienced mild pain in his puncture site comparable to routine PEG procedures. Laboratory studies on postoperative day (POD) 1 revealed mild inflammatory elevation (C‐reactive protein 2.1 mg/dL, white blood cell count 11,190/µL). On POD 2, inflammatory markers progressively increased (C‐reactive protein 11.0 mg/dL, white blood cell count 11,410/µL) despite stable liver enzymes and hemoglobin, raising concern for complications.

**FIGURE 1:**
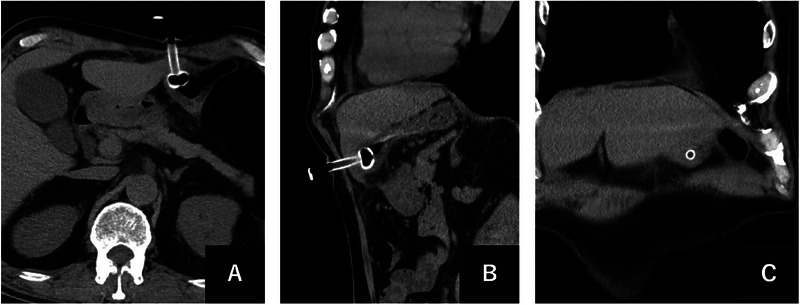
Computed tomography imaging on postoperative day 2 demonstrating transhepatic percutaneous endoscopic gastrostomy tube placement. (A) Axial computed tomography (CT) image showing the 24‐Fr gastrostomy tube penetrating through the thin parenchyma of the hepatic lateral segment before entering the stomach. (B) Sagittal reformatted CT image clearly demonstrating the transhepatic course of the gastrostomy catheter from the abdominal wall through the thin hepatic parenchyma into the gastric lumen. (C) Coronal reformatted CT image showing the gastrostomy tube surrounded by hepatic parenchyma, providing clear visualization of the transhepatic penetration and confirming the inadvertent placement through the hepatic lateral segment.

CT revealed inadvertent transhepatic catheter placement through the hepatic lateral segment with very thin (approximately 10 mm) parenchyma (Figure 1A–C). Concerns arose about potential injury to hepatic arteries, hepatic veins, portal veins, or bile ducts because the large caliber of the gastrostomy tube was penetrating the hepatic parenchyma.

The multidisciplinary team, including gastroenterologists, interventional radiologists, and hepatobiliary pancreatic surgeons, convened to discuss management options. Delayed removal was considered after fistula formation, but immediate removal was chosen due to concerns of hepatic laceration from the tube crossing the thin hepatic parenchyma with respiratory movement. Additionally, minimally invasive removal was prioritized to enable continuation of chemoradiotherapy without delays. To prevent hemorrhage and biliary leakage, embolization by GS for the tract closure was performed collaboratively by gastroenterologists and interventional radiologists, with surgeons on call for emergency surgical intervention if required.

**FIGURE 2:**
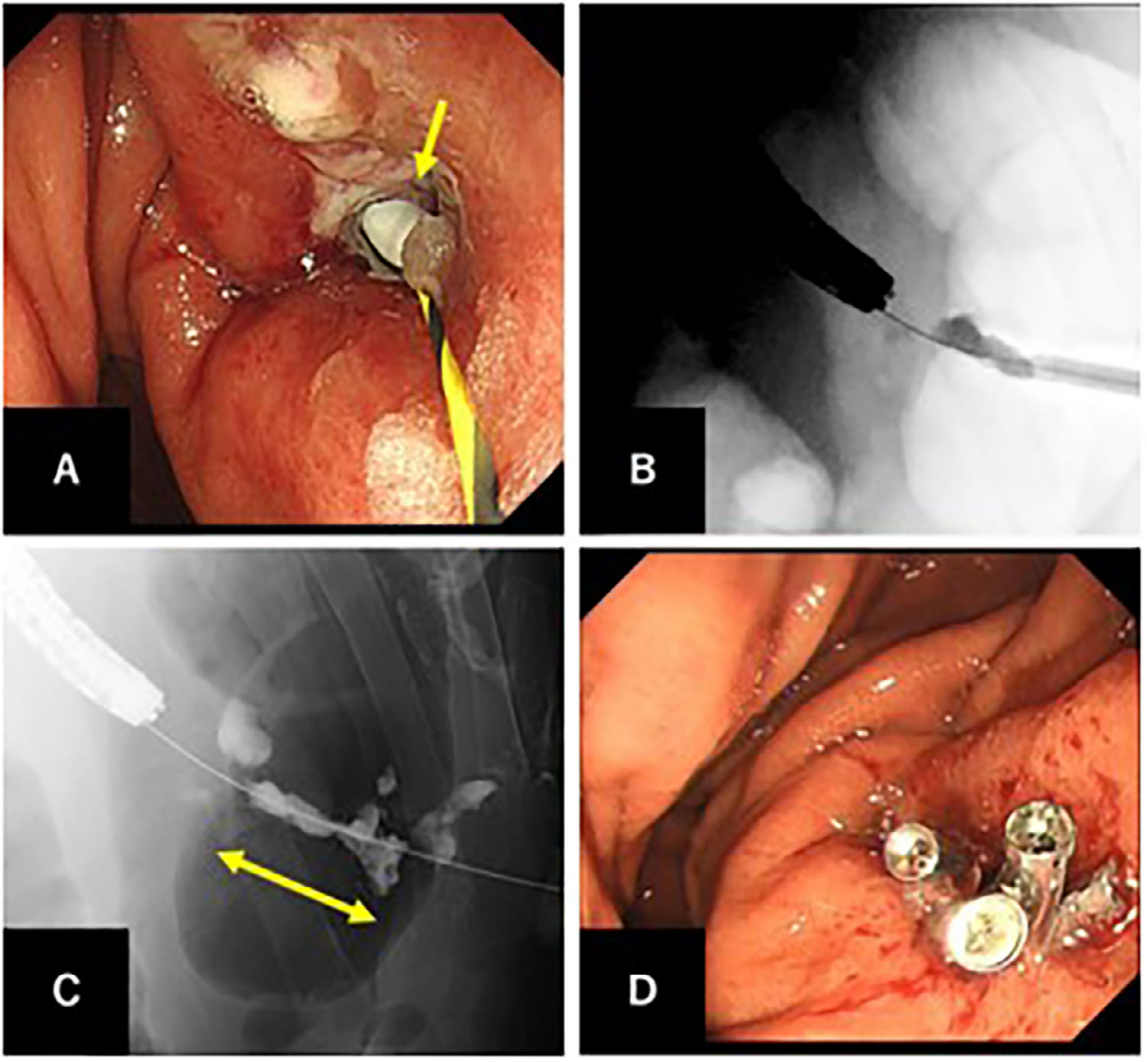
Procedural images demonstrating gelatin sponge tract embolization technique. (A) Endoscopic view showing the 16‐Fr introducer sheath (yellow arrow) inserted over the wire through the gastric wall into the hepatic tract. The pull‐through technique is established for emergency access. (B) Fluoroscopic image during gelatin sponge injection while simultaneously withdrawing the sheath over the wire, demonstrating the embolization process under concurrent endoscopic and fluoroscopic guidance. (C) Fluoroscopic image after gelatin sponge embolization showing complete tract occlusion from the gastric wall through the hepatic parenchyma to the subcutaneous tissue (yellow arrow indicates the embolic material distribution). The guidewire remains in place to maintain tract access. (D) Endoscopic view after guidewire removal showing multiple endoscopic clips applied to close the gastric wall entry site, preventing embolic material displacement into the gastric lumen.

**FIGURE 3:**
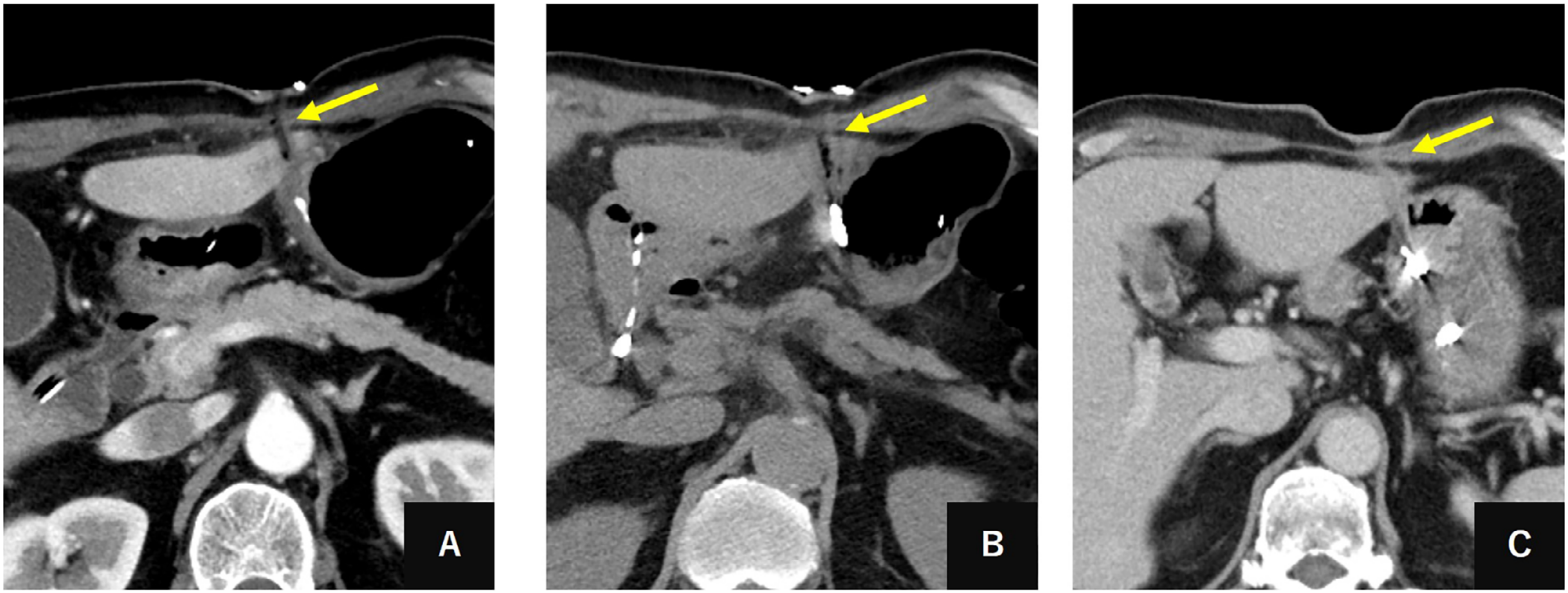
Serial computed tomography imaging demonstrating gelatin sponge tract embolization and healing progression. (A) Axial computed tomography (CT) image 1 day after gelatin sponge embolization showing the embolic material within the hepatic tract with residual gas artifacts (yellow arrow) from the gelatin sponge mixture. (B) Axial CT image 3 days post‐embolization demonstrating persistent gelatin sponge material in the hepatic parenchyma with continued gas artifacts (yellow arrow), confirming successful tract occlusion. (C) Axial CT image 1 month after embolization showing excellent tract resolution with complete absorption of the gelatin sponge material (yellow arrow). The hepatic parenchyma demonstrates normal healing with no evidence of complications.

Initially, a guidewire was inserted through the gastrostomy tube and captured using an endoscopic snare to establish a pull‐through technique. This dual‐access approach was specifically designed to provide emergency tract access for rapid catheter or balloon insertion in the case of massive bleeding during tube removal. After securing safety measures, the gastrostomy tube was carefully removed under direct visualization. Fortunately, only mild bleeding was observed, allowing for the percutaneous insertion of a 16‐Fr sheath over the wire into the tract (Figure 2A), which was considered to be wide enough to inject sufficient embolic material. Highly viscous embolic material was prepared by thoroughly mixing two pieces of GS (Sponzel; LTL Pharma Co., Ltd., Tokyo, Japan) with 4 mL of contrast medium (mixing ratio 2:4 mL) and fragmenting through syringe pumping, creating a paste‐like consistency suitable for tract occlusion. Under concurrent fluoroscopic and endoscopic visualization, this embolic material was carefully injected through the 16‐Fr sheath while simultaneously withdrawing the sheath over the wire (Figure 2B), progressively embolizing the entire tract from the gastric wall through the hepatic parenchyma to the subcutaneous tissue to achieve complete occlusion (Figure 2C). Approximately 2 mL of embolic material was injected. Success was defined as complete tract opacification with contrast stasis within the tract on fluoroscopy. After confirming complete hemostasis, the guidewire was removed. To prevent displacement of embolic material into the gastric lumen or outside of the skin, the gastric wall fistula was closed with endoscopic clips (Figure 2D), and the skin entry site was sealed with dressing material. Serial CT showed no bleeding or biliary fistula formation (Figure 3A–C). The procedure is documented in the Supporting Information video (Video S1). The patient recovered uneventfully with uninterrupted chemoradiotherapy, successfully managed with an ED tube for two months during cancer treatment. CT at six months showed complete tract resolution without residual abnormalities. Surgical gastrostomy was performed six months later for post‐radiation pharyngeal stricture.

## 3 Discussion

Transhepatic PEG placement may present with elevated inflammatory markers and mild symptoms [6], though some cases may remain asymptomatic [2], making diagnosis challenging. This emphasizes the importance of careful postoperative monitoring, particularly when procedural difficulties occur.

Based on previous case reports, transhepatic PEG placement occurs when anatomical factors lead to liver interposition between the gastric and abdominal walls [2, 3, 4, 5, 6]. While our patient had no obvious risk factors such as hepatomegaly, patient movement likely contributed to suboptimal placement, highlighting the importance of adequate sedation and proper technique.

Our staged management approach prioritized minimally invasive intervention under local anesthesia over one‐staged surgical management. The procedure enabled uninterrupted chemoradiotherapy, whereas surgical gastrostomy would have required treatment suspension for at least 2–3 weeks for postoperative recovery. For patients at high risk for general anesthesia, this technique may represent a valuable alternative to immediate surgical intervention.

We propose criteria for this technique, although clear definitions remain challenging to establish. Favorable factors include thin parenchyma (as in our case, ≤10 mm), hemodynamic stability, and interventional expertise with surgical backup. Conservative management may be considered for thick parenchyma and asymptomatic cases. Immediate surgery is indicated for instability, active bleeding, or severe infection.

GS as embolic material offers significant advantages. As a biocompatible, absorbable agent, it provides temporary tract occlusion while allowing healing [7, 8]. GS creates viscous consistency, ensuring tract conformity. Literature supports GS effectiveness with success rates of 98–99% [8, 9]. This represents the first documented use of large‐caliber hepatic tract closure after PEG‐related complications.

Endoscopic clipping closure of the gastric entry site is essential for preventing embolic displacement and ensuring hemostasis. This technique can be performed without dedicated interventional radiology expertise if embolic preparation is mastered.

Given the novel nature of this approach, detailed informed consent was obtained from the patient and family. We explained this as a minimally invasive fistula embolization technique widely used in portal vein catheterization and percutaneous transhepatic biliary drainage. Alternative treatments, including conservative management and surgical intervention, were discussed. Conservative management posed ongoing hepatic laceration risk, leading to the selection of this minimally invasive approach with TAE and surgical backup immediately available.

Prevention remains important for PEG‐related complications. Established safety protocols, including proper positioning, gastric insufflation, and transillumination verification, are essential [1, 10]. In our case, patient agitation caused the complication despite standard measures. When difficulties arise, alternative approaches or postponement should be considered.

This single case report provides limited evidence for clinical adoption. Future multi‐center prospective registries collecting standardized data on anatomical factors, treatment approaches, and outcomes are essential. Accumulated cases will enable comparative studies to establish evidence‐based patient selection criteria and management algorithms. This report demonstrates the feasibility of this novel approach for this extremely rare complication.

## Funding

The authors have nothing to report.

## Conflicts of Interest

The authors declare no conflicts of interest.

## Ethics Statement

Informed consent for publication, including radiologic and endoscopic images, was obtained from the patient.

## Supporting information




**Supporting Video 1**: Gelatin sponge tract embolization technique for safe removal of transhepatic percutaneous endoscopic gastrostomy tube.
Supporting Information
